# Risk Factors of Overweight and Obesity Related to Diet and Disordered Eating Attitudes in Adolescent Girls with Clinical Features of Polycystic Ovary Syndrome

**DOI:** 10.3390/jcm9093041

**Published:** 2020-09-21

**Authors:** Małgorzata Mizgier, Grażyna Jarząbek-Bielecka, Justyna Opydo-Szymaczek, Natalia Wendland, Barbara Więckowska, Witold Kędzia

**Affiliations:** 1 Dietetic Department, Faculty of Physical Culture in Gorzów Wlkp., Poznan University of Physical Education, 66-400 Gorzów Wielkopolski, Poland; 2 Department of Perinatology and Gynecology, Division of Developmental Gynecology and Sexology, Poznan University of Medical Sciences, 60-535 Poznan, Poland; grajarz@ump.edu.pl (G.J.-B.); witold.kedzia@poczta.fm (W.K.); 3 Department of Pediatric Dentistry, Chair of Pediatric Dentistry, Poznan University of Medical Sciences, 60-812 Poznan, Poland; jopydo@ump.edu.pl (J.O.-S.); nataliakryszan@interia.pl (N.W.); 4 Department of Computer Science and Statistics, Poznan University of Medical Sciences, 60-806 Poznan, Poland; basia@ump.edu.pl

**Keywords:** polycystic ovary syndrome, hyperandrogenism, menstrual irregularities, adolescent girls, disordered eating attitudes, obesity, diet

## Abstract

**Background:** We aimed to find the difference between girls with clinical features of Polycystic ovary syndrome (PCOS), divided into two groups: Overweight/obesity (Ov/Ob) and normal weight (N), related to diet, disordered eating attitudes (DEA), metabolic and hormonal differences, and to identify the risk factors of being overweight or obese. **Methods:** Seventy-eight adolescents with PCOS, aged 14–18 years, were divided into Ov/Ob and N groups. Patients underwent blood tests for determination of follicle-stimulating hormone (FSH), luteinizing hormone (LH), total testosterone, DHEA-S, estradiol, of sex hormone-binding globulin (SHBG), fasting glucose, insulin, Homeostatic Model Assessment of Insulin Resistance (HOMA-IR), and lipid profile. Nutrition was evaluated using a 3-day food record. To examine the level of DEA, the Eating Attitudes Test-26 (EAT-26) was used. We defined an EAT-26 score ≥20 as positive for DEA. Logistic regression was carried out to identify the independent predictors of being overweight and obese. **Results:** An increase of 10 g in plant protein intake decreased the probability of being overweight and of obesity (OR = 0.54; *p* = 0.036). EAT-26 score ≥20 was correlated with a 7-fold (OR = 6.88; *p* = 0.02) increased odds of being overweight or of obesity. **Conclusion:** Being overweight and obesity in adolescents with PCOS may be associated with DEA and the type and amount of protein intake.

## 1. Introduction

Polycystic ovary syndrome (PCOS) is the most common cause of hyperandrogenism in girls and women. According to the commonly used Rotterdam criteria, the incidence of PCOS in women ranges from 3% to 26%, and in adolescents it is about 8% [1,2,3]. Clinically hyperandrogenism is manifested by severe acne, scanty or infrequent menstruations, and male hirsutism. Skin changes in the form of acanthosis nigricans, clitoridomegaly, lowering of the voice pitch, depressive syndromes, increase in libido, or reduced sex drive are also often visible. Laboratory tests show borderline or slightly elevated testosterone, androstenedione, and dehydroepiandrosterone sulfate (DHEA-S) levels. Ultrasound examination of the ovaries often reveals their multifollicular structure and increased volume [4]. The initial diagnosis of PCOS can be made in adolescence. Apart from endocrine and reproductive disorders, metabolic changes are often observed in PCOS patients: increased insulin concentration, insulin resistance (IR), and even type 2 diabetes. Moreover, lipid disorders are present. Weight loss is an important component of the management of adolescent patients with PCOS. Obese adolescents with PCOS have a more severe metabolic and hormonal profile than normal-weight adolescents with PCOS. Obese girls usually have earlier menarche. They experience an increased production of androgens caused by too much conversion of hormones in adipose tissue or stimulated by hyperinsulinemia, their excessive production in the ovaries and adrenal glands. The reduced concentration of sex hormone-binding globulin (SHBG) may also cause the pool of free hormones to be higher than in lean girls. Due to these disorders, obese girls remain at increased risk of menstrual disorders, hirsutism, and of developing polycystic ovary syndrome [4,5,6]. In particular, visceral obesity and excess adipose tissue exacerbate IR dyslipidemia, and hormonal disorders [6,7,8,9].

Eating disorders (ED) are mental disorders defined by abnormal eating habits that negatively affect a person’s physical or mental health. The most common eating disorder is binge eating disorder [10,11]. Among all the screening tools used to assess disordered eating attitudes, the EAT is the most commonly used because of its reliability and repeatability in detecting eating disorders in the general population [11,12]. It has been proven that in women with PCOS, ED are more common [13,14,15]. The presence of hirsutism increases the predisposition to ED, which may be associated with lower self-esteem in patients [16]. There are also studies that confirm the more frequent occurrence of disordered eating attitudes (DEA) in patients with an abnormal body mass index (BMI) [17,18,19]. Other studies, however, do not confirm this relationship [12,20]. Therefore, further research is needed to identify the causal relationship between body weight and the occurrence of DEA, as well as to check whether they occur more often in patients with PCOS and, at the same time, with excess body weight.

The aim of this project is to show the differences between the group of adolescent girls at the same age, with normal body weight, and those with overweight and obesity, with clinical features of hyperandrogenism characteristic of PCOS, related to diet (such as animal and plant protein intake, mono-, polyunsaturated and saturated fats, and carbohydrate intake), DEA prevalence, metabolic changes concerning the lipid and carbohydrate profile, hormonal differences concerning sex hormones, and selected fetal and anthropometric factors. We also aimed to identify the factors that increase the risk of being overweight and of obesity among adolescents with clinical features of PCOS, related to diet and DEA.

## 2. Experimental Section

The protocol was approved by the Bioethics Committee at the Poznan University of Medical Sciences (resolution no. 553/18). All adolescent girls and their parents gave their informed consent for inclusion before they participated in the study. This cross-sectional study was conducted in accordance with the Declaration of Helsinki.

### 2.1. Participants

Seventy-eight Caucasian adolescent girls, patients at the Gynecology and Perinatology Medical Clinic at the Gynecology and Obstetrics Hospital of Poznan University of Medical Sciences, aged 14–18 years, participated in the research conducted in 2018–2020. All subject were at least two years after menarche and sought medical diagnosis for the signs of clinical and biochemical abnormalities.

The criteria for the patient’s inclusion were based on the 2003 Rotterdam criteria [21,22,23], with the presence of at least two of the following:

- clinical and/or biochemical hyperandrogenism (hirsutism with moderate to severe acne, and/or elevation of serum total testosterone or free testosterone,
- oligoovulation (based on oligomenorrhea defined as bleeding episodes occurring less than 8 times per year or secondary amenorrhea),
- polycystic ovarian picture in an ultrasound examination (at least 12 follicles in each ovary each measuring 2–9 mm in diameter and/or ovarian volume >10 mL).

The exclusion criteria were as follows: any systemic disease or medications of continuous use, the use of hormonal therapy or antibiotics in the past three months, Cushing syndrome, congenital adrenal hyperplasia, hyperprolactinemia suggestive of pituitary adenoma, thyroid dysfunction, diabetes, and androgen-secreting tumors.

The girls selected for the study were divided into two groups: normal weight—N group (n = 45, mean age ± SD = 16.29 ± 1.08) and overweight/obese— Ov/Ob group (n = 33, mean age ± SD = 15.73 ± 1.66).

### 2.2. Medical Evaluation

After admission to the hospital, during two days of hospital stay, the participants were referred for the gynecological consultation in the hospital outpatient department. The examiner (G.J.B.) verified the presence of any symptoms of hormonal disturbances. Participants answered questions about their menstrual cycle regularity and age of menarche. The physical examination included an evaluation of the skin for signs of virilization, such as acne and hirsutism, ultrasound examination (transabdominal) and blood tests. The procedure of a transabdominal ultrasound has been described previously [24]

### 2.3. Anthropometric and Body Composition Assessment

All measurements were conducted after a 12 h fast. All the patients had their height, body mass, and waist circumference (WC) measured. Weight was measured to the nearest 0.1 kg using SECA 899 digital medical scales. Height was measured to the nearest mm with a SECA 217 stadiometer attached to the scales. The patients were asked to remove their shoes and outer garments. WC was measured to the nearest 0.1 mm using Gulick anthropometric tape between the lower border of rib margin and the upper border of iliac crest (WC-mid). Diagnosis and classification of overweight and obesity was based on BMI, according to World Health Organisation (WHO) for children aged 5–19 years overweight and obesity correspond to BMI-for-age greater than 1 standard deviation and 2 standard deviations above the WHO growth reference median, respectively [25].

Body composition was assessed with a Tanita MC 780 body composition analyzer, which uses bioelectrical impedance analysis (BIA method). In this model, the accuracy of measurement for the individual components, including adipose tissue, is 100 g. The measurements of fat mass (FM) were expressed as a percentage (%) and kilograms (kg). Full details of this method have been described elsewhere [26,27].

### 2.4. Biochemical Parameters

Blood samplings and a gynecological examination with ultrasound were performed in the early follicular phase (days 3–5), apart from patients with secondary amenorrhea.

Hormonal and biochemical parameters, including follicle-stimulating hormone (FSH), luteinizing hormone (LH), total testosterone, estradiol, DHEA-S, SHBG, fasting glucose and fasting insulin, total cholesterol (TC), high-density lipoprotein cholesterol (HDL-C), and triglycerides (TG) were measured in the morning after overnight fasting.

Biochemical analyses were performed in the central hospital laboratory. Plasma glucose was measured by the enzymatic method with hexokinase. Insulin, FSH, LH, total testosterone, 17-β-estradiol, DHEA-S, SHBG were measured by the electrochemiluminescence (ECLIA) immunoassay method (Elecsys) (Roche Diagnostics GmbH, Mannheim, Germany). Plasma TC, HDL-C, TG levels were determined by the enzymatic colorimetric method (Roche Diagnostics GmbH, Mannheim, Germany). Low-density lipoprotein cholesterol (LDL-C) was calculated using Friedewald’s formula (LDL-C [mg/dL] = TC-HDL-C-TG/5).

The free testosterone level was calculated from total testosterone and SHBG levels using the online free testosterone calculator available on the ISSAM Website (www.issam.ch/freetesto.htm). The free androgen index was calculated as 100 × (T/S), where T is total testosterone in nanomoles per liter and S is SHBG in nanomoles per liter.

Homeostasis model assessment of insulin resistance (HOMA-IR) was applied using the following formula: HOMA-IR = fasting insulin (μU/mL) × fasting glucose (mmol/l)/22.5 [28,29].

The results were compared to the age and sex-specific laboratory reference ranges and the literature data [30,31].

### 2.5. The Data from the Child’s Health Book

The data were collected from the child’s health book regarding newborn body weight and term of delivery. The incidence of macrosomia (body weight over 4000 g) or low birth weight, LBW (body weight less than 2500 g) was assessed on the basis of the newborn’s body weight [32,33]. The full term of delivery (term births) was assessed for deliveries that occur between 38–42 weeks of pregnancy, while preterm births were defined as those deliveries between 23 weeks and 37 weeks [34].

### 2.6. Nutrition Evaluation (Evaluation of the Diet)

The girls’ eating habits (a balanced diet) were evaluated by a *registered dietitian* (M.M.). For this purpose, the current quotation method from three consecutive days was used.

The method used in this study to assess eating habits was the qualitative-quantitative method of current quotation (Three-day food record), from three consecutive days, including two business days and one holiday day [35]. This method was used to assess the diet of patients at the beginning of the study. The 3-day food record method aims to record the amount of all food products needed to prepare meals and meals consumed by the subject. To properly assess the size of the portion consumed, respondents used photos from the album of products and dishes of the National Food and Nutrition Institute in Warsaw [36]. The Aliant computer program (Cambridge Diagnostics) was used to calculate the energy and nutritional value of daily food rations (DFR) of the women studied. Standard losses associated with the technical and culinary treatment were included in the calculations.

In each case, the analyzed daily food intake was compared with the current, individual daily nutritional and energy requirement in accordance with the Human Nutrition Standards of the Food and Nutrition Institute, taking into account the availability of energy at the level recommended for girls [37].

### 2.7. Eating Behavior

Eating behavior was assessed with a standard EAT-26, a self-administrated questionnaire used worldwide [11,12]. The questionnaire consists of 26 questions related to attitudes and beliefs, behavior related to eating, the perception of appearance, and body weight. The questions were grouped into three sub-scales that facilitate the determination of disrupted dietary attitudes. The first scale, slimming, which is dominated by bulimic behavior, assesses dissatisfaction with the shape and appearance of the body. The second scale, labeled ‘bulimia and eating control’ contains features of bulimic behavior and behavior related to compulsive eating and increased body weight. The third scale called ‘oral control’ identified behavior and beliefs related to eating and stemming from forced control and restrictions. This scale assesses anorexia and reduced body weight. The participant replies to a question by selecting one response: always, usually, often, sometimes, rarely, never. In the first 25 questions, the answer ‘always’ is worth 3 points, ‘usually’, 2 points, and ‘often’, 1 point. In question 26, the answer ‘never’ is worth 3 points, ‘rarely’, 2 points, and ‘sometimes’, 1 point. A total score of 20 or more points indicates DEA [12]. The authors of EAT-26 included five questions related to inducing vomiting, the use of cathartics, feeling of having no control over food intake, excessive physical activity, and significant weight loss over a short period. In order to determine whether a participant is susceptible to disordered eating behavior, it sufficed that they selected an answer in the EAT-26 questionnaire related to such a pathological behavior regardless of the frequency [11,12,38,39,40].

### 2.8. Statistical Analyses

Quantitative data describing two independent groups and meeting the normal distribution were compared using the Student’s t-test or the Student’s t-test with the Cochran–Cox correction, when the variances of the groups being compared were significantly different. The Mann–Whitney test was used when the assumption of normal distribution was not met. The dichotomous data were compared with the chi-square test or the Fisher’s exact test when the Cochran conditions for using the chi-square test were not met. For the ordered category data, the Cochran–Armitage trend test was used. The study of the normal distribution was performed using the Shapiro–Wilk test, and the comparison of the variance of variables between the groups was performed using the Fisher–Snedecor test. The groups are described as mean ±SD (standard deviations), medians and quartiles, as well as numbers and percentages respectively. Logistic regression (univariate and multivariate) was carried out to identify the independent predictors of being overweight and obese. A significance level of 0.05 was adopted in all analyses. The analyses were performed in the PQStat v1.8.0 program.

## 3. Results

### 3.1. Anthropometric, Clinical, Metabolic, and Hormonal Parameters

In the group of overweight and obese patients, a statistically significant difference was demonstrated in relation to the second study group in terms of WC (*p* < 0.001) and the content of fat mass expressed as a percentage (FM%) (*p* < 0.01). In this group, a significantly lower age of menarche was also found (*p* = 0.026) (Table 1). There were no statistically significant differences between the groups in terms of birth weight [g], but in the group of overweight and obese girls, there were over 2.5 times more cases of macrosomia. There were also no significant differences between the groups regarding at-term births, while in the overweight and obese group, there were 4 times fewer cases of preterm births (Table 1). The patients also differed significantly in terms of systolic (SBP) and diastolic blood pressure (DBP), and in both cases, the significance of the differences was *p* < 0.001 (Table 1).

Girls who were overweight and obese differed from lean patients in terms of metabolic and hormonal changes, namely, a significantly lower HDL concentration (*p* < 001) and higher TG concentration (*p* = 0.08) were observed in them. Moreover, statistically significantly higher levels of fasting glucose (*p* = 0.012) and fasting insulin (*p* < 0.001) were found. HOMA-IR index scores were also significantly higher in the overweight and obese group (*p* < 0.001) (Table 2).

In the Ov/Ob group, a higher value of the FAI index (*p* < 0.01), free testosterone (*p* < 0.01), and DHEA-S concentration (*p* = 0.028) was observed, and a lower concentration of SHBG (*p* < 0.001) than in patients from the N group (Table 2).

### 3.2. Energy and Nutritional Value of Daily Food Intake and DEA

The analysis of the daily food intake of the patients proved that there were no significant differences between the groups in terms of the average energy value and nutritional value of the daily food intake. Nevertheless, the energy value of the diet in patients from the Ov/Ob group was over 200 kcal lower than in the second study group. Overweight and obese patients consumed over 8 g less fat, including both saturated, mono- and polyunsaturated, and almost 30 g less carbohydrates. At the same time, it was shown that a low glycemic index (GI) of the diet was almost three times less frequent in overweight and obese patients than in lean patients, and the daily consumption of fiber in the Ov/Ob group was almost 4.5 g lower. Protein consumption was also about 6 g lower in the overweight and obese group, while this difference was mainly due to a lower consumption of vegetable protein (Table 3).

An increase of 10 g in plant protein intake per day decreased the probability of being overweight and of obesity, OR = 0.54, significantly *p* = 0.04. The reduction in the probability of abnormal body weight was maintained regardless of age, OR = 0.53, *p* = 0.04, and predisposition to DEA (OR = 0.52, *p* = 0.04 (Table 3).

No statistical significance was found for the remaining dietary components that were tested. On the other hand, the odds ratio increased almost 4-fold, OR = 3.7; *p* = 0.06 for the consumption of products with a medium GI and 2-fold, OR = 2, *p* = 0.6 for the consumption of products with a high GI. After adjusting for age, the increase in both cases decreased slightly, while after adjusting for age and the presence of DEA, the odds ratio increased more than 4-fold (Table 3)

As shown in the data presented in Table 2, the risk of presenting DEA is more than 5 times more common in overweight and obese patients (2 (4.4%) vs. 8 (24.2%), *p* = 0.015). The mean value of the sum of points obtained in the EAT-26 in the N group and the Ov/Ob group differed and amounted to 9.27 (± 5.67) vs. 13.49 (± 9.54), while no statistical significance in this respect was demonstrated (Table 2). It was found, however, that patients with DEA had an almost 7-times higher probability of being overweight and obese than people without such a predisposition (OR = 6.88, *p* = 0.02). After taking into account the age of the respondents, the risk increases more than 14-fold (OR = 14.74, *p* = 0.003) (Table 3).

## 4. Discussion

Most studies conducted previously assumed that PCOS was a disease of women of reproductive age. Therefore, only in recent years have there been several publications on the clinical picture of PCOS in adolescent girls, taking into account the multifactorial etiology of PCOS, including environmental factors.

A number of factors have been identified in girls associated with an increased risk of PCOS. Fetal factors include macrosomia and low birth weight in girls born to mothers with excess body weight. In adolescents, they include premature puberty, metabolic syndrome, and obesity [41]. The presence of obesity may exacerbate reproductive and metabolic disorders in the form of insulin resistance, type 2 diabetes, and abnormalities in blood pressure regulation [13,42].

The aim of our study was to check whether there are significant differences between the groups of girls aged 14–18 years, with normal and abnormal body weight, with clinical features of PCOS, in terms of sex hormones, metabolic parameters related to lipid and carbohydrate metabolism, anthropometric parameters, and fetal factors. The aim of this study was also to assess the differences in the diet between lean and overweight and obese patients, as well as in the occurrence of DEA. As the reports of recent studies confirm that women with PCOS also have an increased risk of their occurrence [14,15,43]. The main goal of our study was to check whether the energy value of the diet and macronutrient intake, as well as the presence of DEA, are associated with an increased ratio of overweight and obesity in adolescents.

### 4.1. Anthropometric, Metabolic, Hormonal Parameters, and Fetal Factors

In our study, we did not find statistically significant differences between the groups in terms of birth weight [g], but in the group of overweight and obese girls, there were twice as many cases of macrosomia. As already mentioned, in previous studies, excessive birth weight of girls born to mothers with excess body weight predisposed them to PCOS [41]. In the study of Qiao et al., there is also a description of a positive relationship between excessive birth weight and a higher probability of obesity in girls [44]. In the overweight and obese group, the age of menarche was significantly lower (*p* = 0.026). Moreover, there was a significant correlation between menarche age and the risk of being overweight and of obesity (OR = 0.63, *p* = 0.02), with the odds ratio slightly increasing after adjusting for age (OR = 0.65, *p* = 0.04) and age and DEA (OR = 0.72, *p* = 0.1) (Table 3).

An earlier study by Carroll et al. conducted on women with PCOS at the age of 18–45 also found a strong correlation between body weight expressed in BMI and the age of menarche. Chromosome 6 variant rs7759938-T was associated with the earlier age of menstruation [45]. An increased risk of metabolic changes, including the occurrence of IR, is widely reported in the literature on PCOS patients [46]. In particular, obese adolescent girls with polycystic ovary syndrome have increased rates of cardiovascular disease risk [47]. In PCOS, disorders related to lipid indices are more common: a lower concentration of HDL and a higher concentration of TG and LDL-C [22]. An important determinant of metabolic risk in women with PCOS is the high content of adipose tissue, especially visceral adipose tissue [48].

Comparing the groups studied in terms of metabolic parameters, we observed a significant difference in overweight and obese girls compared to the slim ones in terms of WC (*p* < 0.001) and the content of FM% (*p* < 0.01) (Table 1). The girls with overweight and obesity differed from slim patients in terms of a lower HDL concentration (*p* < 0.001) and higher TG concentration (*p* = 0.08) (Table 2). In the overweight and obese group, statistically significantly higher levels of fasting glucose (*p* = 0.012) and fasting insulin (*p* < 0.001) were also found. The HOMA-IR related to IR was also significantly higher in the Ov/Ob group (*p* < 0.001) (Table 2). Patients with normal and abnormal body weight also differed significantly in terms of SBP and DBP. In both cases, the significance of the differences was *p* < 0.001, to the disadvantage of obese and overweight patients (Table 2).

Excessive weight and/or insulin resistance has been related to the development of metabolic syndrome in adolescents with PCOS. It is not clear however what role hyperandrogenism has on the development of metabolic syndrome or its role on those metabolic parameters associated with metabolic syndrome. Forrester-Dumont et al. showed that the metabolic profile in adolescent girls with PCOS is not affected by either the presence of hyperandrogenism or the degree of hyperandrogenism [49]. Other studies confirmed the relationship between the concentration of testosterone, DHEA-S, and SHBG and the pathophysiology of PCOS and metabolic disorders [50,51,52,53]. A strong influence of androgens on the cardio-metabolic risk was confirmed, indicating that androgens should be considered an important determinant of women’s health [53]. In our study, the testosterone concentration in the Ov/Ob group did not differ significantly compared to the N group, while in the Ov/Ob group, a higher FAI value was observed (*p* < 0.01). In this group, there was also a higher concentration of DHEA-S (*p* = 0.028) and a lower concentration of SHBG (*p* < 0.001) compared to the lean ones. Wildman et al. suggest that variation in the adipose hormones resulting from lower SHBG levels, and possibly greater androgenicity, may contribute to susceptibility for metabolic and cardiovascular outcomes. Regulators of adipose tissue hormones remain incompletely understood, but may include sex hormones [52].

### 4.2. Energy and Macronutrients Intake

Literature data on the effect of specific dietary components on overweight and obesity in PCOS patients are very limited and inconclusive. On the other hand, obesity occurs among 40–70% of young PCOS patients [54]. Earlier studies showed that a negative energy balance (with a caloric deficit of 350 to 1000 kcal/day) is a key factor associated with the reduction in body weight and adipose tissue, as well as the improvement of metabolic and hormonal indices in PCOS patients [55,56,57,58,59]. In our study, overweight and obese patients did not differ significantly from slim girls in terms of their energy intake. The relationship with overweight and obesity in our study may have been caused not so much by the energy value but by its composition, including the quality of consumed carbohydrates and proteins.

We have shown that the low GI, related to the type of carbohydrate consumed, occurred four times less often in overweight and obese patients than in lean patients, and the daily intake of fiber in the Ov/Ob group was almost 4.5 g lower than in the N group. We also show that the probability of overweight and obesity increased almost 4-fold (OR = 3.7; *p* = 0.06) for the consumption of products with a medium GI and 2-fold (OR = 2, *p* = 0.6) for the consumption of products with a high GI. After adjusting for age, the increase in both cases decreased slightly, while after adjusting for age and DEA, the OR increased more than 4-fold (Table 3). The results of studies described by several other authors suggest a positive effect of low GI on glucose metabolism and an improvement in the hormonal response in women with PCOS, which was associated with weight reduction [55,56,60,61,62,63]. A low GI diet tends to be higher in fiber, and fiber is also important in improving metabolic rates in PCOS, including weight reduction.

Previous studies show that when a 12-month dietary intervention was used with a diet low in energy, low in saturated fat, medium or high in fiber, and with low or high GI, a low GI diet provided a three times’ greater improvement in insulin sensitivity, and, moreover, improved menstrual regularity compared to a conventional low-fat hypocaloric diet. Its positive effect on lipid indices and blood androgen levels was also observed [60]. Wong et al. showed that a low GI diet led to a greater reduction in BMI in adolescents with PCOS than those with high GI and medium GI [62]. These researchers also showed improvements in metabolic risk factors, including insulin sensitivity [63].

The results of previous studies also showed that high protein content in the diet has an impact on the reduction of body weight and adipose tissue. Sørensen et al. demonstrated that a high-protein and low-carbohydrate diet significantly reduced body weight, FM, WC, and fasting glucose compared to a low-protein and high-carbohydrate diet [64]. Dong et al. suggested in an RCT meta-analysis that a 6-month long high-protein, moderate and low-carbohydrate diet may have a beneficial effect on weight loss, HbA1c, and blood pressure in patients with impaired glucose metabolism [65]. On the other hand, Toscani et al. did not observe any differences in the reduction of body weight, WC, and FM by comparing the effects of high-protein and moderately low-carbohydrate diets as well as low-protein, high-carbohydrate diets [66]. The results of Kasim-Karakas et al.’s study comparing the effects of different types of proteins and different carbohydrate content showed a greater loss of body weight and fat mass in a moderately low-carbohydrate diet with a high content of whey protein [67]. The beneficial effects of a higher-protein diet on body composition and weight loss are likely related to the greater effect of protein (compared to carbohydrate and fat) on inducing postprandial thermogenesis [68,69]. Protein also increases satiety, possibly due to the increased production of cholecystokinin. The stimulus to increase the secretion of cholecystokinin are mainly the products of protein digestion. This hormone weakens intestinal motility and has an anti-hunger effect [70,71,72]. Although some studies show that high-protein diets can have a number of positive health effects, including maintaining lean body mass while losing weight and body fat, improving glycemic control, and improving other risk factors for cardiovascular disease such as blood pressure, it is still unclear whether these effects are due to higher protein intake or lower carbohydrate intake. In addition, studies do not indicate which type of protein intake has a more beneficial effect (plant or animal protein). Our research shows that protein intake in the Ov/Ob group was about 6 g lower than in the N group, while this difference was mainly due to a lower consumption of plant protein. At the same time, we showed that an increase in the consumption of plant protein by 10 g/day doubles the probability of overweight and obesity in teenage patients (OR = 0.54, *p* = 0.04). The reduction in the probability of excess body weight is maintained regardless of age, OR = 0.53, *p* = 0.04 and DEA, OR = 52, *p* = 0.04 (Table 3).

In our studies, overweight and obese patients also consumed over 8 g less fat, including both saturated fatty acids (SFA), monounsaturated fatty acids (MUFA), and polyunsaturated fatty acids (PUFA). However, we did not show any significant correlation between overweight and obesity and dietary fat intake. However, in previous clinical trials in patients with PCOS, low-carbohydrate, high-fat diets have been shown to reduce fasting insulin levels and improve insulin sensitivity [73,74,75]. It was also demonstrated that a high-fat, moderately low-carbohydrate eucaloric diet resulted in a greater reduction in body fat, including visceral tissue, compared to the control diet [76,77,78].

### 4.3. Eating Behavior

Based on the EAT-26 results, we have shown that DEA occurs four times more often in patients with PCOS and simultaneously with overweight and obesity. The difference between the groups was statistically significant (*p* = 0.015). The mean value of the sum of EAT-26 points in the N group and the Ov/Ob group was 9.27 (± 5.67) vs. 13.49 (± 9.54), respectively (Table 3). Moreover, we found that patients with DEA are seven times more likely to be overweight and obese than people without DEA (OR = 6.88, *p* = 0.02). After taking into account the age of the respondents, this probability increased over 14 times (OR = 14.74, *p* = 0.003).

In previous studies, it was found that the risk of obesity, as well as the severity of hirsutism in patients with PCOS, was associated with eating disorders [16,17]. Rouzitalab et al. also showed significant correlations between body weight and WC and the results of EAT-26, in the group of women aged 18 to 25 years. Body weight and WC in women proved to be good markers associated with an increase in the EAT-26 score [19]. Moreover, it has been shown that not only overweight but also female gender were both the factors associated with DEA [79,80]. Rodriguez et al. showed that the use of certain dietary strategies reduces the likelihood of developing DEA. In studies conducted among athletes, the introduction of an appropriate nutritional program reduced the risk of unhealthy habits that could turn into eating disorders [18]. This finding shows that certain dietary strategies can be useful in preventing eating disorders if they are diagnosed early enough.

### 4.4. Strengths and Weaknesses

There are some limitations to our study that should be mentioned. The EAT-26 questionnaire used is not a diagnostic but a screening tool. Another limitation was that our study included a relatively small group of patients. Moreover, the diagnosis of PCOS was based on the Rotterdam criteria [21,22,23], while some experts recommend the use of more stringent criteria in adolescent patients as the symptoms of typical puberty may mimic the symptoms typical/characteristic of PCOS [80]. To limit misdiagnosis, we chose patients who were at least two years after menarche and those who were admitted to the hospital because of persistent problems with the menstrual disorders and clinical symptoms of hyperandrogenism [81].

On the other hand, this is the first study to show that DEA is associated with a higher likelihood of excess body weight in PCOS patients. To our knowledge, this is also the first study to identify plant protein as a dietary factor that may reduce the likelihood of excess body weight in adolescent patients with PCOS.

## 5. Conclusions

In our study, overweight and obese patients did not differ significantly from slim girls in terms of their energy, animal and plant protein intake, mono-, polyunsaturated and saturated fats, and carbohydrate intake, but the low GI, related to the type of carbohydrate consumed, occurred four times less often in overweight and obese patients than in lean patients.

We have shown that DEA occurs four times more often in patients with PCOS and simultaneously with overweight and obesity. The difference between the groups was statistically significant.

Comparing the groups studied in terms of metabolic parameters, we observed a significant difference in overweight and obese girls compared to the slim ones in terms of WC [cm], body mass [kg], and the content of FM [%] and FM [kg]. The girls with overweight and obesity differed from slim patients in terms of a lower HDL concentration and higher TG concentration. In the overweight and obese group, statistically significantly higher levels of fasting glucose and fasting insulin were also found. The HOMA-IR related to IR was also significantly higher in the Ov/Ob group. Patients with normal and abnormal body weight also differed significantly in terms of SBP and DBP. In both cases, the significance of the differences was *p* < 0.001, to the disadvantage of obese and overweight patients

In our study, we did not find statistically significant differences between the groups in terms of birth weight [g], but in the group of overweight and obese girls, there were twice as many cases of macrosomia.

The testosterone concentration in the Ov/Ob group did not differ significantly compared to the N group, while in the Ov/Ob group, a higher FAI value was observed. In this group, there was also a higher concentration of DHEA-S and a lower concentration of SHBG compared to the lean ones.

The original idea of our research is to consider potential factors related to diet and eating behavior that may increase the risk of excess body weight in girls with PCOS.

Our study demonstrates the importance of early screening for DEA in PCOS patients, because certain dietary strategies can be used to prevent them [18]. Furthermore, we show that DEA can increase the likelihood of being overweight and obese.

Moreover, our results show that the quality of the diet, in particular the quality of carbohydrates and protein, may play a role in modulating body weight in PCOS patients.

The effect of specific dietary components on the prevalence of overweight and obesity in girls with PCOS should be further investigated, especially in dietary intervention studies. Such results would make it possible to support the treatment of patients with clinical features of PCOS with dietary therapy. This is important, because diet is one of the risk factors for high BMI, although it is also one of those modifiable factors that can be used as independent therapy in patients with PCOS.

## Figures and Tables

**Table 1:** Comparison of the baseline parameters including age, selected anthropometric, and clinical parameters between groups.

	N Group n = 45	Ov/Ob Group n = 33	*p*-Value
Age (years)			0.200
mean (sd)	16.29 (1.08)	15.73 (1.66)	
median (25–75%)	16 (16–17)	16 (14–17)	
Body height (m)			0.213
mean (sd)	1.65 (0.07)	1.67 (0.05)	
median (25–75%)	1.65 (1.61–1.71)	1.67 (1.64–1.69)	
Body weight (kg)			<0.001
mean (sd)	54.56 (8.34)	84.31 (13.02)	
median (25–75%)	54.3 (50.2–62.1)	82.1 (77.5–92.2)	
Waist Circumference [cm]			<0.001
mean (sd)	70.69 (6.51)	93.09 (10.9)	
median (25–75%)	69 (67–74)	91 (87–98)	
Fat mass [%]			<0.001
mean (sd)	19.73 (7.47)	33.72 (7.41)	
median (25–75%)	18.8 (16.1–26.1)	33.5 (27.7–38.3)	
Fat mass [kg]			<0.001
mean (sd)	8.02 (5.36)	21.25 (10.71)	
median (25–75%)	7.1 (4.4–10.2)	18.5 (11.7–29.3)	
Menarche (years)			0.026
mean (sd)	12.56 (1.21)	11.82 (1.36)	
median (25–75%)	12 (12–13)	12 (11–13)	
Birth weight classification			0.152
Low birth weight	4 (8.9%)	2 (6.1%)	
Normal	38 (84.4%)	25 (75.8%)	
Macrosomia	3 (6.7%)	6 (18.2%)	
Term of delivery			0.228
Term births	39 (86.7%)	32 (97%)	
Preterm births	6 (13.3%)	1 (3%)	
SBP [mmHg]			<0.001
mean (sd)	103.29 (9.04)	116.94 (11.19)	
median (25–75%)	103 (98–109)	116 (110–123)	
DBP [mmHg]			<0.001
mean (sd)	67.67 (8.3)	75.39 (9.63)	
median (25–75%)	67 (62–73)	76 (69–81)	

SBP—systolic blood pressure [mmHg]; DBP—diastolic blood pressure [mmHg].

**Table 2:** Comparison of measured parameters between groups.

	N Group n = 45	Ov/Ob Group n = 33	*p*-Value		N Group n = 45	Ov/Ob Group n = 33	*p*-Value
TC [mg/dL]			0.765	Energy intake [kcal]			0.164
mean (sd)	161.13 (30.79)	157.64 (23.96)		mean(sd)	1740.33 (538.67)	1533.69 (392.63)	
median (25–75%)	158.1 (140.3–175.2)	159 (135.2–176.4)		median (25–75%)	1663.5 (1467.7–1884.7)	1592.5 (1234.8–1738.8)	
LDL-C [mg/dL]			0.407	Protein [g]			0.701
mean (sd)	83.37 (27.22)	86.42 (20.77)		mean (sd)	73.7 (29.74)	67.68 (18.34)	
median (25–75%)	83.3 (61.1–97.7)	82.3 (68.1–102.1)		median (25–75%)	67.1 (53.6–86.6)	67.9 (52.2–79.7)	
HDL-C [mg/dL]			<0.001	Fat [g]			0.231
mean (sd)	60.21 (11.53)	48.84 (8.14)		mean (sd)	64.75 (23.6)	56.48 (22.14)	
median (25–75%)	58 (52.9–68.3)	48.5 (43.5–54.4)		median (25–75%)	61.3 (49.9–79.2)	57.9 (42.8–70.4)	
TG [mg/dL]			0.008	Carbohydrates [g]			0.088
mean (sd)	87 (33.92)	111.88 (43.81)		mean (sd)	225.49 (82.8)	196.06 (48)	
median (25–75%)	82.3 (59.9–108.3)	104.2 (74.7–154.4)		median (25–75%)	216.9 (193.8–235.5)	198.5 (177.3–221.3)	
Fasting glucose [mg/dL]			0.012	Dietary fiber [g]			0.281
mean (sd)	87.25 (5.53)	90.98 (7.13)		mean (sd)	20.13 (12.77)	15.81 (6.75)	
median (25–75%)	87 (84–90.5)	90.65 (85.95–96.3)		median (25–75%)	16.1 (11.5–24.9)	14.3 (10.9–18.5)	
Fasting insulin [mU/mL]			<0.001	Plant protein [g]			0.058
mean (sd)	12.23 (6.74)	20.31 (8.16)		mean (sd)	25.16 (13.46)	19.42 (6.29)	
median (25–75%)	11.2 (8.29–14.87)	19.23 (15.57–26.05)		median (25–75%)	22 (17.4–29.7)	19.1 (14.8–22.7)	
HOMA-IR			<0.001	Animal protein [g]			0.934
mean (sd)	2.63 (1.63)	4.79 (2.18)		mean (sd)	41 (20.8)	40.68 (14.32)	
median (25–75%)	2.35 (1.75–3.15)	4.26 (3.33–5.62)		median (25–75%)	36.8 (29–49.2)	37.8 (33–52.6)	
Total testosterone [ng/dl]			0.125	Saccharose [g]			0.189
mean (sd)	0.5 (0.23)	0.57 (0.21)		mean (sd)	36.63 (23.01)	31.57 (22.65)	
median (25–75%)	0.47 (0.38–0.57)	0.6 (0.4–0.7)		median (25–75%)	31.7 (22.7–46.8)	26.7 (18.9–39.3)	
Free testosterone				SFA og. [g]			0.104
mean (sd)	6.47 (4.63)	10.65 (4.32)	<0.001	mean (sd)	25.29 (9.88)	21.6 (9.65)	
median (25–75%)	5.4 (3.9–8)	10.9 (6.6–14)		median (25–75%)	24.2 (18.8–29.4)	23.2 (14.6–26.7)	
FAI			<0.001	MUFA og. [g]			0.551
mean (sd)	3.37 (2.9)	6.77 (3.25)		mean (sd)	22.01 (9.37)	20.14 (9.02)	
median (25–75%)	2.54 (1.6–4.2)	7.2 (3.6–9.6)		median (25–75%)	19.9 (15.8–26.6)	22.1 (12.6–24.5)	
DHEA-S [µmol/l]			0.028	PUFA og. [g]			0.675
mean (sd)	6.64 (2.66)	8.11 (3.07)		mean (sd)	9.04 (5.03)	7.86 (3.38)	
median (25–75%)	6.31 (5.25–8.02)	8.21 (5.7–11.21)		median (25–75%)	7.1 (5.2–11.5)	7.8 (5.2–10.4)	
SHBG [nmol/l]			<0.001	Cholesterol [mg]			0.915
mean (sd)	73.66 (39.49)	33.81 (14.89)		mean (sd)	256.08 (156.91)	236.56 (98.15)	
median (25–75%)	66.11 (44.27–98.24)	32.61 (21.86–40.67)		median (25–75%)	232.7 (162.3–298.7)	210.9 (180.7–280.5)	
LH [mlU/mL]			0.336	Glycemic Index (GI)			0.121
mean (sd)	10.34 (8.06)	13.03 (10.41)		low (≤55)	12 (26.7%)	3 (9.1%)	
median (25–75%)	7.85 (5.17–16.66)	11.18 (4.22–16.93)		moderate (55–70)	31 (68.9%)	29 (87.9%)	
FSH [mlU/mL]			0.288	high (≥70)	2 (4.4%)	1 (3%)	0.015
mean (sd)	5.36 (2.29)	4.71 (2.25)		DEA			
median (25–75%)	5.37 (4.22–6.44)	4.94 (3.16–6.64)		No	43 (95.6%)	25 (75.8%)	
Estradiol [pg/mL]			0.288	Yes	2 (4.4%)	8 (24.2%)	0.069
mean (sd)	72.94 (93.82)	130.45 (312.69)		EAT-26 score			
median (25–75%)	46.7 (34.48–69.92)	53.56 (39.54–84.43)		mean (sd)	9.27 (5.67)	13.49 (9.54)	

DHEA-S—dehydroepiandrosterone, SHGB—sex hormone-binding globulin, LH—luteinizing hormone sulfate, FSH—follicle-stimulating hormone, TC—total cholesterol, LDL-C—low-density lipoprotein cholesterol, HDL-C—high-density lipoprotein cholesterol, TG—triglycerides, HOMA-IR—Homeostatic Model Assessment of Insulin Resistance; DEA—disordered eating attitudes; EAT–Eating Attitude Test.

**Table 3:** The odds ratio of Eating Attitudes Test-26 (EAT-26) score, glicemic index (IG), energy and nutrients intake, and the risk factors of being overweight or of obesity.

	The Odds Ratio of Overweight/Obesity					
	OR (95% CI)	*p*-Value	AOR (95% CI)	*p*-Value *	AOR (95% CI)	*p*-Value **
EAT-26 score	1.08 (1.01–1.15)	0.023	1.09(1.02–1.16)	0.009	1.09(1.02–1.16)	0.009
DEA [Yes]	6.88 (1.35–34.97)	0.02	14.74 (2.49–87.08)	0.003	-	-
DEA [No]	reference		reference		reference	
menarche	0.63 (042–0.93)	0.02	0.65 (0.44–0.98)	0.04	0.72 (0.47–1.09)	0.121
Energy [kcal] [kcal]	1 (1–1)	0.075	1 (1–1)	0.085	1 (1–1)	0.069
Energy [100 kcal]	0.9 (0.8–1.01)		0.9 (0.79–1.01)		0.87 (0.75–1.01)	
Protein [g]	0.99 (0.97–1.01)	0.309	0.99 (0.97–1.01)	0.359	0.99 (0.97–1.01)	0.384
Protein [10 g]	0.9 (0.75–1.1)		0.91 (0.75–1.11)		0.91 (0.73–1.13)	
Fat [g]	0.98 (0.96–1)	0.125	0.98 (0.96–1)	0.116	0.98 (0.96–1)	0.113
Fat [10 g]	0.85 (0.69–1.05)		0.84 (0.68–1.04)		0.82 (0.64–1.05)	
Carbohydrates [g]	0.99 (0.98–1)	0.085	0.99 (0.98–1)	0.11	0.99 (0.98–1)	0.089
Carbohydrates [10 g]	0.93 (0.85–1.01)		0.93 (0.85–1.02)		0.91 (0.82–1.01)	
Fiber [g]	0.96 (0.91–1.01)	0.092	0.96 (0.91–1.01)	0.159	0.96 (0.91–1.01)	0.133
Plant protein [g]	0.94(0.89–1)	0.036	0.94 (0.88–1)	0.039	0.94 (0.88–1)	0.044
Plant protein [10 g]	0.54(0.3–0.96)		0.53 (0.29–0.97)		0.52 (0.27–0.98)	
Animal protein [g]	1 (0.97–1.02)	0.937	1 (0.97–1.02)	0.935	1 (0.97-1.03)	0.931
Animal protein [10 g]	0.99 (0.77–1.27)		0.99 (0.77–1.28)		1.01 (0.77–1.33)	
Saccharose [g]	0.99 (0.97–1.01)	0.337	0.99 (0.97–1.01)	0.326	0.98 (0.96–1.01)	0.185
Saccharose [10 g]	0.9 (0.73–1.12)		0.9 (0.72–1.12)		0.85 (0.67–1.08)	
SFA [g]	0.96 (0.91–1.01)	0.108	0.96 (0.91–1.01)	0.095	0.96 (0.91–1.01)	0.128
SFA [10 g]	0.67 (0.41–1.09)		0.65 (0.39–1.08)		0.65 (0.38–1.13)	
MUFA [g]	0.98 (0.93–1.03)	0.374	0.98 (0.93–1.03)	0.38	0.97 (0.92–1.03)	0.344
MUFA 10 [g]	0.8 (0.48–1.32)		0.79 (0.47–1.33)		0.76 (0.43–1.35)	
PUFA [g]	0.94 (0.84–1.05)	0.25	0.94 (0.84–1.05)	0.27	0.92 (0.81–1.04)	0.187
Cholesterol [mg]	1 (1–1)	0.527	1 (1–1)	0.597	1 (1–1)	0.96
Cholesterol [10 mg]	0.99 (0.96–1.02)		0.99(0.96–1.03)		1 (0.96–1.04)	
Glycemic Index [low (≤55)]	reference		reference		reference	
Glycemic Index [moderate (55–70)]	3.74 (0.96–14.62)	0.058	3.13 (0.78–12.56)	0.108	4.26 (0.91–19.91)	0.065
Glycemic Index [high (≥70)]	2 (0.13–30.16)	0.617	1.86 (0.12–28.19)	0.655	4.38 (0.25–77.61)	0.314

EAT–Eating Attitudes Test; DEA–Disordered Eating Attitudes; * The odds ratio adjusted for age; ** The odds ratio adjusted for age and EAT-26 score.
